# Aberrant monocyte responses predict and characterize dengue virus infection in individuals with severe disease

**DOI:** 10.1186/s12967-017-1226-4

**Published:** 2017-05-31

**Authors:** Yean K. Yong, Hong Y. Tan, Soe Hui Jen, Esaki M. Shankar, Santha K. Natkunam, Jameela Sathar, Rishya Manikam, Shamala D. Sekaran

**Affiliations:** 1 Centre of Excellence for Research in AIDS (CERiA), Department of Medicine, Faculty of Medicine, University of Malaya, Kuala Lumpur, Malaysia; 2 Department of Medical Microbiology, Faculty of Medicine, University of Malaya, Lembah Pantai, 50603 Kuala Lumpur, Malaysia; 3 Hospital Tengku Ampuan Rahimah, Persiaran Tengku Ampuan Rahimah, Klang, Selangor Malaysia; 4 Clinical Hematology Laboratory, Department of Hematology, Hospital Ampang, Ampang, Selangor Malaysia; 5 Department of Trauma and Emergency Medicine, University Malaya Medical Centre, Kuala Lumpur, Malaysia; 6 Division of Infection Biology and Microbiology, Department of Life Sciences, School of Basic and Applied Sciences, Central University of Tamil Nadu (CUTN), Neelakudi Campus, Tiruvarur, India

**Keywords:** Dengue, Severe dengue, Dengue with warning signs, IL-18, sCD14, LBP, Monocyte activation, Microbial translocation

## Abstract

**Background:**

Currently, several assays can diagnose acute dengue infection. However, none of these assays can predict the severity of the disease. Biomarkers that predicts the likelihood that a dengue patient will develop a severe form of the disease could permit more efficient patient triage and allows better supportive care for the individual in need, especially during dengue outbreaks.

**Methods:**

We measured 20 plasma markers i.e. IFN-γ, IL-10, granzyme-B, CX3CL1, IP-10, RANTES, CXCL8, CXCL6, VCAM, ICAM, VEGF, HGF, sCD25, IL-18, LBP, sCD14, sCD163, MIF, MCP-1 and MIP-1β in 141 dengue patients in over 230 specimens and correlate the levels of these plasma markers with the development of dengue without warning signs (DWS−), dengue with warning signs (DWS+) and severe dengue (SD).

**Results:**

Our results show that the elevation of plasma levels of IL-18 at both febrile and defervescence phase was significantly associated with DWS+ and SD; whilst increase of sCD14 and LBP at febrile phase were associated with severity of dengue disease. By using receiver operating characteristic (ROC) analysis, the IL-18, LBP and sCD14 were significantly predicted the development of more severe form of dengue disease (DWS+/SD) (AUC = 0.768, *P* < 0.0001; AUC = 0.819, *P* < 0.0001 and AUC = 0.647, *P* = 0.014 respectively). Furthermore, we also found that the levels of VEGF were directly correlated and sCD14 was inversely correlated with platelet count, suggesting that the endothelial activation and microbial translocation may played a role in pathogenesis of dengue disease.

**Conclusions:**

Given that the elevation IL-18, LBP and sCD14 among patients with severe form of dengue disease, our findings suggest a pathogenic role for an aberrant inflammasome and monocyte activation in the development of severe form of dengue disease.

**Electronic supplementary material:**

The online version of this article (doi:10.1186/s12967-017-1226-4) contains supplementary material, which is available to authorized users.

## Background

Dengue virus (DENV) infection remains the most common arthropod-borne viral infection worldwide [1, 2], with a recent global estimate suggesting that ~390 million clinical DENV cases occur annually [3]. Nearly ~75% of the global DENV disease burden is concentrated across South East Asia and Western Pacific [4], and the incidence had been steadily increasing over the past decade at the rate of ~threefold from 1.2 million in 2008 to 3.2 million in 2015 [3]. In 2015 alone, 111,000 DENV infection cases were reported from Malaysia [3]. Though the mortality rates due to dengue is relatively low, the widespread distribution of DENV has led to significant morbidity across the country warranting a strong demand for hospital beds, finances and personnel thereby diverting recourses from other medical areas [5]. However, not all DENV infections develop complications requiring hospitalization. According to the WHO classification 2009, dengue has been classified as with or without warning signs and severe dengue [6] based on clinical symptoms and laboratory tests. Nonetheless, the initial clinical symptoms of dengue cannot distinguish between mild dengue from severe disease, and warning signs may only develop eventually as the disease progresses [7, 8]. Furthermore, these warning signs usually occur only a day prior to clinical deterioration [9, 10] rendering effective interventions challenging [11]. Hence, there is a pressing need to identify biomarkers that will triage dengue patients into those that are most or least likely to develop severe forms of dengue disease, allowing best supportive care prioritised to individuals in need, especially during epidemics [12].

Cytokine storm has been shown to affect vascular endothelial permeability [13], and has also been associated with dengue haemorrhagic fever (DHF) and dengue shock syndrome (DSS) [14–16], as per the classical dengue disease classification. However, the role of cytokine storm and its association with the WHO 2009 classification i.e. dengue without warning signs, dengue with warning signs and severe dengue have seldom been studied. One recent study showed that products associated with microbial translocation, i.e. lipopolysaccharide (LPS) and the monocyte activation marker soluble CD14 (sCD14) were elevated among patients with severe dengue disease. This suggests that apart from viral factors, cytokine storm may also be at least in part due to the elevation of LPS in plasma and perhaps other danger-associated molecule patterns (DAMPs). Here, we investigated plasma markers including cytokines, chemokines as well as other inflammatory mediators that predict the severity of dengue disease with respect to the WHO 2009 classification. Our results indicate that markers associated with inflammasome activation (IL-18), microbial translocation, monocyte activation (sCD14) and LPS binding protein (LBP) were among the best predictors in clinical DENV infection.

## Methods

### Study design and population

The longitudinal study was conducted on patient specimens archived from a descriptive cohort previously reported [7]. Patients were recruited from the emergency department and dengue wards of two hospitals situated in the Klang Valley, Malaysia: Ampang Hospital (Ampang, Selangor) and Tengku Ampuan Rahimah Hospital (Klang, Selangor) from June 2010 to April 2011. The criteria for inclusion in the study were as follows: (i) individuals above the age of 14 years (ii) dengue infection confirmed by either one of the following methods including RT-PCR, NS1 antigen test, a minimum of fourfold increment of IgM and/or IgG titer, and/or an IgM and/or IgG conversion in paired serum (iii) availability of biological specimens. In the parental study, blood was drawn at three time-points i.e. febrile, defervescence and convalescence from each patient, span down and stored in −80 °C freezer until used. Because the aim of the present study was to identify biomarkers that predict severity of dengue disease when patients first presented, only specimens collected during the febrile and defervescence phases were included in the investigation. Later, all patients were grouped into dengue without warning signs (DWS−), dengue with warning signs (DWS+) and severe dengue (SD) according to WHO 2009 dengue classification (elaborated in Additional file 1: Table S1). Informed consent/assent for all patients were obtained in the parental study. This study was approved by the ethical committees of all the hospitals and laboratories involved: University Malaya Medical Center (782.90), Ampang Hospital (NMRR-10-683-6420), Tengku Ampuan Rahimah (NMRR-10-683-6420).

### Quantification of cytokines, chemokines and inflammatory mediators

We evaluated twenty biomarkers in patients with confirmed dengue virus infection and compared them among DWS−, DWS+ and SD. Biomarkers were broadly chosen from three pathways implicated in dengue pathogenesis: (i) inflammation [monocyte chemoattractant protein-1 (MCP-1), macrophage inflammatory proteins-1b (MIP-1b), CXCL6, CXCL8, IP-10, CX3CL1, sCD25, granzyme B, IFN-γ, IL-10, hepatocyte growth factor (HGF), soluble (s)CD163, IL-18, RANTES and macrophage migration inhibitory factor (MIF)] [17–22], (ii) endothelial activation [vascular endothelial growth factor (VEGF), intercellular adhesion molecule 1 (ICAM-1) and vascular cell adhesion molecule 1 (VCAM-1)] [23, 24] and (iii) microbial translocation [sCD14 and lipopolysaccharide binding protein (LBP)] [25, 26].

The plasma levels of MCP-1, MIP-1b, CXCL6, CXCL8, IP-10, CX3CL1, sCD25, granzyme B, IFN-γ, IL-10, HGF, sCD163, VEGF, ICAM-1 and VCAM-1 were measured human Luminex screening assay (R&D Systems, Inc. Minneapolis, MN, USA); whilst IL-18, IL-18BPa, sCD14 RANTES and macrophage migration inhibitory factor (MIF) were measured using Quantikine ELISA kit (R&D Systems, Inc. Minneapolis, MN, USA). The free circulating IL-18 (IL-18 molecules that have not bound to its natural occurring inhibitor, IL-18BP) was estimated using the law of mass action [27], with IL-18 and IL-18BP interaction at ratio of 1: 1 and dissociation constant (Kd) of 0.4 nmol/l [28, 29]. All analytes were measured according to the manufacturer’s protocol. The details of plasma volume used and dilution factors were provided in the Additional file 1: Table S2.

### Statistical analysis

The primary aim was to compare biomarkers among the three groups of dengue patients i.e. dengue without warning signs, dengue with warning signs and severe dengue at febrile and defervescence phase. Comparisons of categorical variables were tested using Chi square test, and continuous variables were tested using a non-parametric Kruskal–Wallis test for multiple group comparisons. If P values were <0.05, 3-way comparisons were subsequently performed separately using Mann–Whitney U tests between the 3 patient groups applying the Benjamini–Hochberg correction for multiple comparisons [30].

The predictive power of biomarkers at febrile and defervescence phase was examined using receiver operating characteristic (ROC) analysis. A Wilcoxon matched-pairs test was used for paired analyses for febrile and defervescence phase values. The Spearman rank test was used to compare correlations between continuous variables. Biomarkers associated with more severe form of dengue disease were then evaluated by binary logistic regression followed by adjusted logistic regression. The odds ratio and 95% confidence interval (CI) were estimated.

Statistical analyses were performed using Prism, version 5.02 (GraphPad Software, San Diego, CA). Binary regression was performed using SPSS, version 20 (IBM, Armonk, NY), and the heat map (Fig. 5b) was generated using Plotly (https://plot.ly/). Two-tailed P <0.05 was considered as statistical significance for all test performed and P value <0.05, <0.01, <0.001, <0.0001 by Mann–Whitney U test were marked as *, **, *** and **** respectively. P values that remain statistical significant after Benjamini–Hochberg correction are marked in red.

## Results

### Patients characteristics

Plasma cytokine levels of hundred and forty-one adult patients with laboratory confirmed dengue virus infection were investigated. Patients were classified by the WHO 2009 guidelines into 43 (35%) with ‘‘Dengue without warning signs (DWS−)’’, 92 (65.2%) with ‘‘Dengue with warning signs (DWS+) and 6 with ‘‘Severe dengue (SD)’’. A total of 230 plasma specimens (febrile, n = 126; defervescence, n = 104) were included in this study, of which specimens for 89 (36.1%) patients were paired. Age of patients ranged from 14 to 61 years old with median, interquartile range (IQR) of 25 [21, 34] years old. 68.8% were male. Other demographics and clinical parameters including history of previous dengue disease, comorbidities and days of fever are described in Table 1. There was no significant difference between all the demographic and clinical parameters between the 3 patient groups.

**Table 1:** Patients characteristics at admission

Characteristics	Dengue disease			P value
	DWS−	DWS+	Severe	
Number	43 (30.5%)	92 (65.2%)	6 (4.3%)	141
Age, year	26 (22, 35)	25 (20, 35)	23 (22, 31.5)	0.935
Gender, male	34 (79%)	59 (64.1%)	4 (66.7%)	0.445
History of dengue disease	1 (2.3%)	4 (4.3%)	0 (0%)	0.618
Comorbidities				
Diabetes mellitus	1 (2.3%)	5 (5.4%)	0 (0%)	0.466
Hypertension	3 (7%)	3 (3.2%)	1 (16.7%)	0.451
Chronic kidney disease	0	0	0	–
Ischemic heart disease	1 (2.3%)	0 (0%)	1 (16.7%)	0.536
Congestive heart failure	0	0	0	–
Day of sample collection^a^, days				
Febrile phase	4 (3, 5)	4 (3, 5)	2.5 (1.5, 4.5)	0.247
Defervescence phase	5 (4, 6)	5 (4.5, 6.5)	5 (4.75, 6.5)	0.543

Comparison of demographic and clinical characteristics between dengue patients with and without warning signs and severe dengue. All data are expressed as median (IQR) unless specified. *P* values are calculated by Chi square test for categorical variable and Kruskal–Wallis test for continuous variables

*IQR* interquartile range

^a^Counting from day 1 of fever

### Elevation of plasma levels of IL-18 were associated with dengue with warning signs and severe dengue

**Fig. 1:**
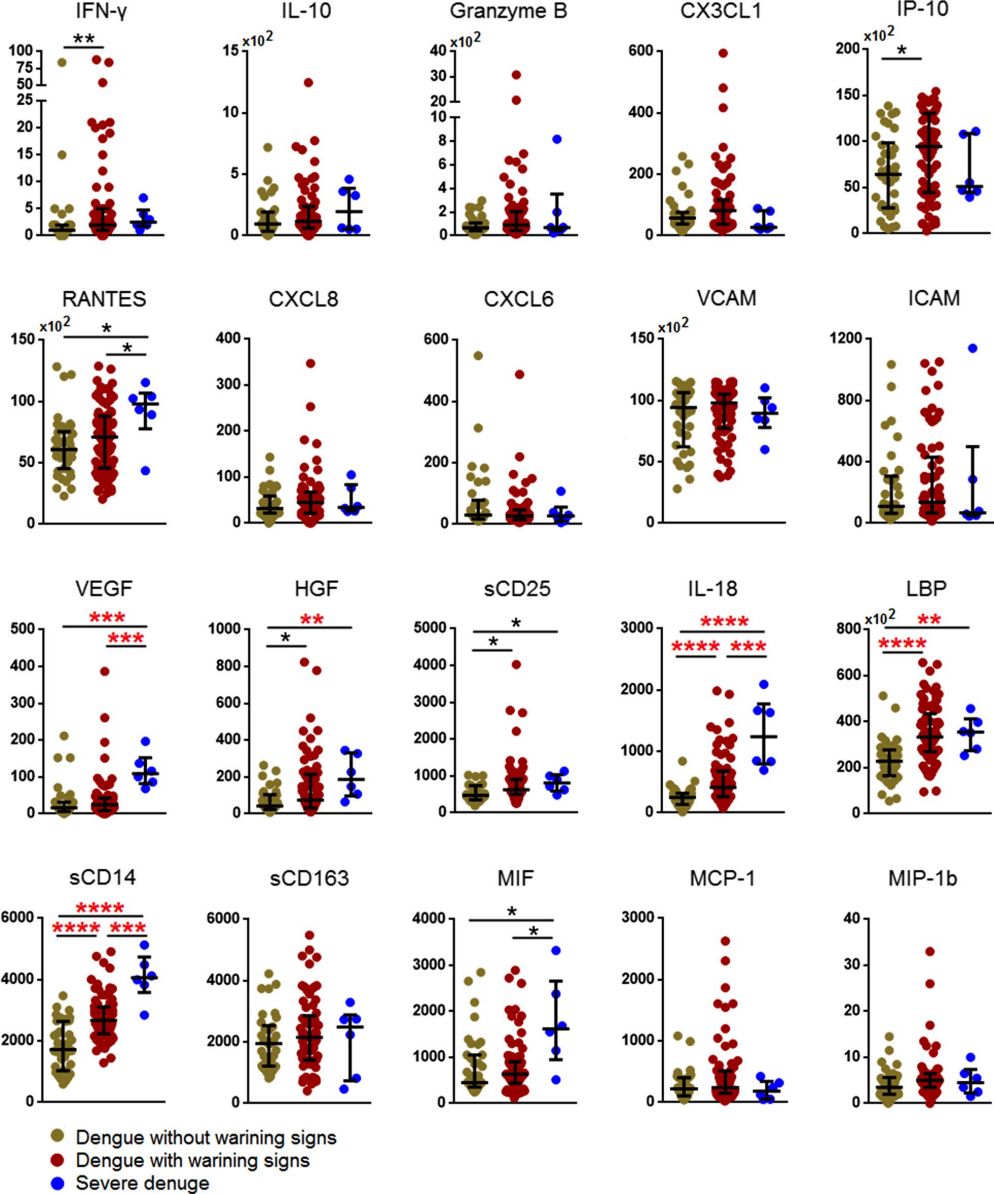
Comparison of plasma levels of biomarkers in dengue patients without warning signs, without warning signs and severe dengue at febrile phase. Levels of biomarkers were compared across the three patient groups by Kruskal–Wallis test. Post hoc Mann–Whitney U tests were then performed for those biomarkers with a Kruskal–Wallis test *P* value of <0.05. *P* value less than 0.05 (significant), where *<0.05, **<0.01, ***<0.001, ****<0.0001. *P* values remain significant after Benjamini–Hochberg correction of multiple comparisons were marked in *red* *

**Fig. 2:**
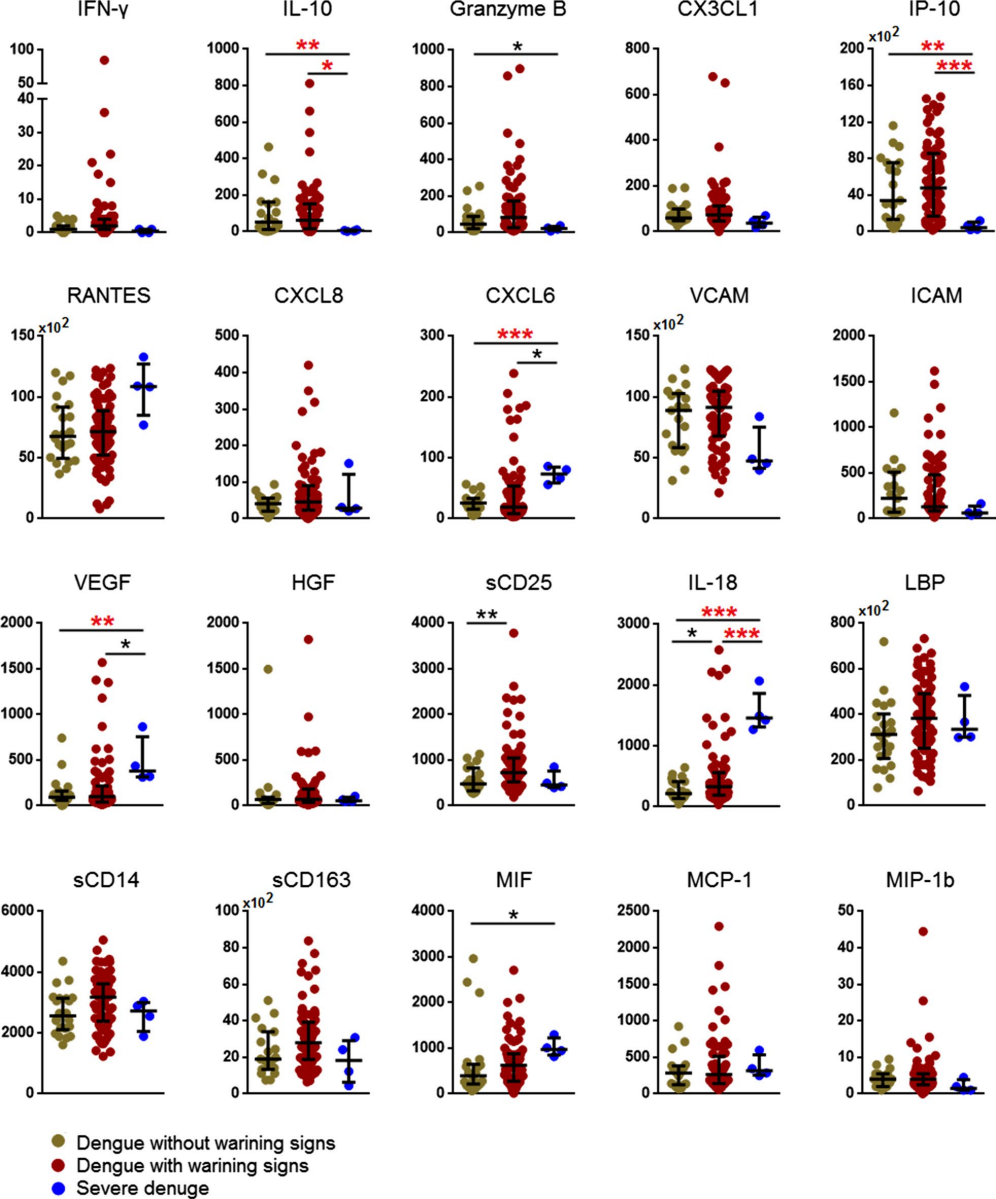
Comparison of plasma levels of biomarkers in dengue patients without warning signs, without warning signs and severe dengue at defervescence phase. Levels of biomarkers were compared across the three patient groups by Kruskal–Wallis test. Post hoc Mann–Whitney U tests were then performed for those biomarkers with a Kruskal–Wallis test *P* value of <0.05. *P* value less than 0.05 (significant), where *<0.05, **<0.01, ***<0.001, ****<0.0001. *P* values remain significant after Benjamini–Hochberg correction of multiple comparisons were marked in *red* *

Comparison of all three study groups in our cohort revealed that patients experiencing SD had the highest plasma levels of IL-18 (median = 1240 pg/ml; IQR = 696.1, 1741.3 pg/ml) followed by DWS+ (median = 414 pg/ml; IQR = 244.7, 687.9 pg/ml) and DWS− (median = 250 pg/ml; IQR = 137, 314.9 pg/ml) at febrile phase (Fig. 1). The differences were significant (*P* < 0.0001). During the defervescence phase, the levels of IL-18 remained higher in patients with SD (median = 1460 pg/ml; IQR = 1159, 1643.2 pg/ml) in comparison with DWS+ (median = 329 pg/ml; IQR = 229.3, 616.3 pg/ml; *P* < 0.001) and DWS− (median = 217 pg/ml; IQR = 137.5, 413.5 pg/ml; *P* < 0.001) (Fig. 2). These association remained significant even after Benjamini–Hochberg correction.

Biological activity of IL-18 on the other hand is regulated by a naturally occurring regulatory protein, IL-18 binding protein (IL-18BP). IL-18BP is a soluble molecule that binds to IL-18 with high affinity, hence preventing IL-18 reaching the cell surface receptors, thereby regulating bioavailability of IL-18 to other lymphocytes [31]. Having showed that plasma levels of IL-18 was associated with the severity of dengue disease, we next assessed the circulating levels of this regulatory molecule and the free circulating IL-18 (fraction of IL-18 that do not bound to IL-18BP). We showed that the IL-18BP levels were not significantly different between the 3 groups at both febrile and defervescence phases, however the free circulating IL-18 levels were significantly higher in SD (median_feb_ = 44 pg/ml; median_def_ = 60 pg/ml) followed by DWS+ (median_feb_ = 12.2 pg/ml; median_def_ = 11.8 pg/ml) and DWS− (median_feb_ = 9.0 pg/ml; median_def_ = 8.2 pg/ml). Furthermore, Spearman analysis between IL-18 and IL-18BP at defervescence phase showed that IL-18 and IL-18BP was better correlated in DWS− (r = 0.645, *P* = 0.0016) patient as compared to DWS+ and SD patients (r = 0.291, *P* = 0.006) (Additional file 2: Figure S1). These data suggest that the IL-18BP may be low in DWS+/SD patients.

### Increase of microbial translocation and monocyte activation marker at febrile phase were associated with severity of dengue disease

**Fig. 3:**
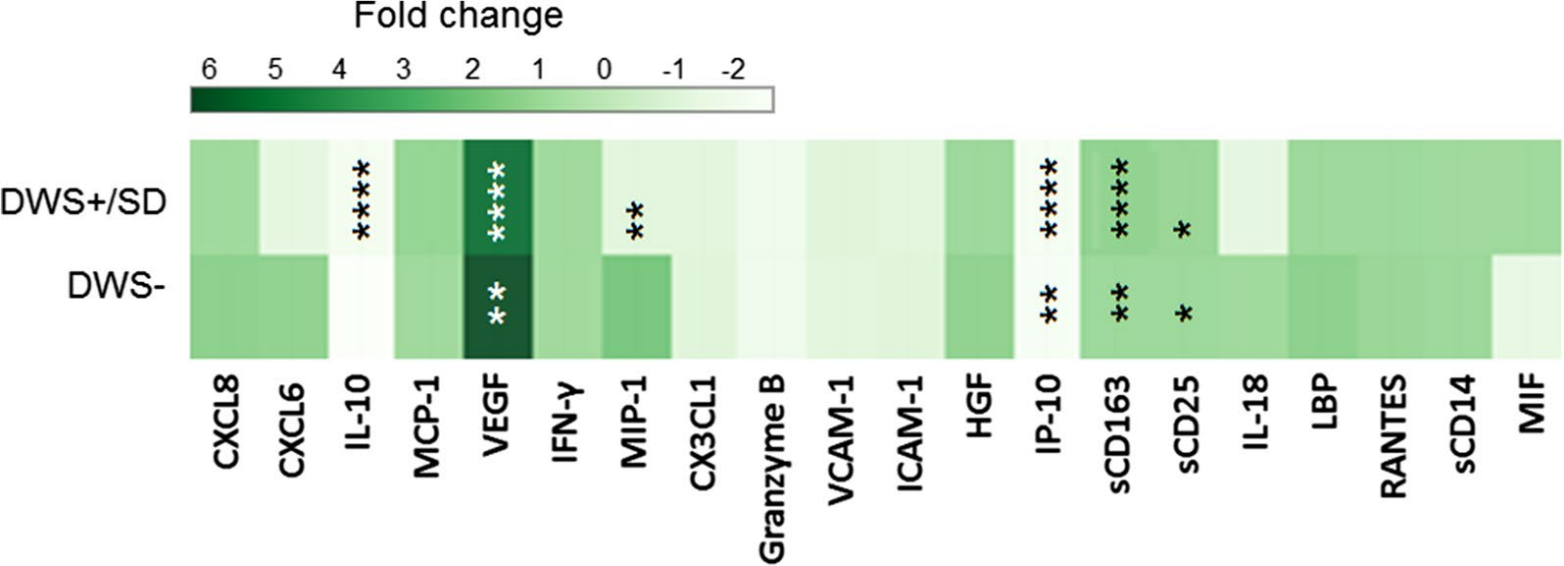
Fold-change of plasma levels of biomarkers in dengue patients without warning signs, without warning signs and severe dengue over the different phases of illness. A Wilcoxon matched-pairs test was used to assess the changes of biomarkers levels from febrile phase to defervescence phase. Fold-change of plasma marker was defined as the median level of each biomarkers at defervescence phase was divided by their respective level at febrile phase. The fold-change was then reflected by the colour scale in the heatmap in *P* value less than 0.05 (significant), where *<0.05, **<0.01, ***<0.001, ****<0.0001

To determine if other biomarkers of monocyte and macrophage activation had any association with the severity of dengue disease, we assessed plasma levels of LPS binding protein (LBP), sCD14, sCD163, MIF, MCP-1 and MIP-1b in the three study groups. We found that the LBP and sCD14 at febrile phase were significantly associated with the severity of dengue disease. Compared to DWS− (median = 23,860 pg/ml), LBP was highest in SD group (median = 35,530 pg/ml, *P* < 0.01) followed by DWS+ (median = 33,346 pg/ml, *P* < 0.0001). A similar pattern was observed with sCD14. Compared to DWS− (median = 1890 pg/ml), sCD14 was also highest in SD+ (median 4230 pg/ml, *P* < 0.0001) followed by DWS+ (median = 2847 pg/ml, *P* < 0.0001) (Fig. 1). These associations remain significant after correction with Benjamini–Hochberg correction. Other monocyte/macrophage markers such as sCD163, MIF, MCP-1 and MIP-1b were not significantly different among the three groups after adjustment for multiple comparisons. Nonetheless, Wilcoxon analysis showed that sCD163 were significantly increased 1.37-fold in DWS+/SD compared to 1.08-fold in DWS− patients (Fig. 3).

### Decreased IP-10 and IL-10 levels as well as increased levels of CXCL6 and VEGF were associated with severe dengue

At defervescence phase, IL-10 and IP-10 were found to be decreased among patients with SD as compared to DWS+ (*P* < 0.05; *P* < 0.001 respectively) and DWS− (both *P* < 0.01); whilst CXCL6 were significantly elevated in patients with SD compared to DWS− (*P* < 0.001). Wilcoxon analysis showed that plasma levels of IL-10 and IP-10 among SD and DWS+ patient were significantly reduced by 2.1-and 2.2-fold respectively (Fig. 3). VEGF levels were also associated with SD whereby its levels at both febrile (*P* < 0.001) (Fig. 1) and defervescence (*P* < 0.05) (Fig. 2) phases were highest among patients with SD as compared to DWS+ and DWS−. VEGF levels at defervescence phase generally followed the same pattern (Fig. 2). Wilcoxon analysis showed that patients with SD and DWS+ had VEGF levels increased 5.2-fold from 25 pg/ml at febrile phase to 132 pg/ml at defervescence phase (Fig. 3).

### Plasma levels of sCD14, LBP, IL-18 predicted the development of dengue with warning signs and severe dengue

**Fig. 4:**
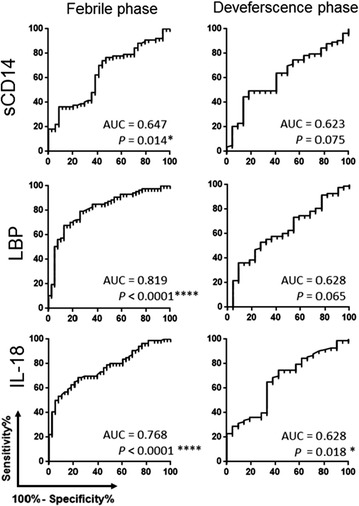
Receiver operating characteristic curves for prediction of dengue with warning signs and severe dengue. The sensitivity and specificity of sCD14, LBP and IL-18 in predicting DWS+/SD were assessed by using receiver operating characteristic curve where *top panel* sCD14, *middle panel* LBP, *bottom panel* IL-18, *left panel* febrile phase, *right panel* defervescence phase. *AUC* are under curve

Using ROC analyses, plasma levels of IL-18 [area under curve (AUC) = 0.768, *P* < 0.0001], sCD14 (AUC = 0.647, *P* < 0.014) and LBP (AUC = 0.819, *P* < 0.0001) at febrile phase were observed to be strong candidate biomarkers for predicting DWS+ and SD. While during defervescence phase, IL-18 was the only marker that predicted DWS+ and SD (AUC = 0.628, *P* < 0.018) (Fig. 4).

To further examine the relationship between heightened biomarkers and the severity of dengue disease, we assessed the relationship of biomarkers that had shown to be significantly elevated in SD and DWS+ in both febrile phase (i.e. IL-18, LBP and sCD14) and defervescence phase (IL-18, CXCL6, IL-10 and IP-10) by using binary regression model controlling for age, gender and platelet counts. Our multivariate model showed that all the three IL-18, LBP and sCD14 markers at febrile phase were independently associated with the more severe form of dengue disease; whilst at defervescence phase, only was IL-18 was found to be independently associated with severity of dengue disease (Table 2).

**Table 2:** Predictor of dengue disease severity

	Univariate		Multivariate	
	Coef. (95% CI)	P value	Coef. (95% CI)	P value
Febrile phase				
Platelet count	0.997 (0.998, 1.005)	0.431	–	–
VEGF	1.005 (0.996, 1.014)	0.259	–	–
IL-18 (per 10 unit)	1.036 (1.015, 1.058)	0.001**	1.032 (1.006, 1.059)	0.017*
LBP (per 1000 unit)	1.131 (1.073, 1.191)	0.0001***	1.106 (1.002, 1.198)	0.013*
sCD14 (per 100 unit)	1.195 (1.114, 1.282)	0.0001***	1.106 (1.022, 1.198)	0.013*
Defervescence phase				
Platelet count	0.998 (0.990, 1.006)	0.694	–	–
IL-18 (per 10 unit)	1.003 (1.001, 1.006)	0.02*	1.003 (1, 1.006)	0.048*
CXCL6	1.01 (0.995, 1.026)	0.205	–	–
IL-10	1 (0.997, 1.003)	0.956	–	–
IP-10	1 (1, 1)	0.517	–	–

Associations of plasma biomarkers at febrile and defervescence phase with the severity of dengue disease. By using binary regression model, variables that showed a significant relationship with the development of dengue with warning signs and severe dengue in univariate model will then be included in the multivariate model. The Hosmer–Lemeshow value for this model was P = 0.743

* P < 0.05

### Plasma levels of VEGF and sCD14 correlated with platelet counts

**Fig. 5:**
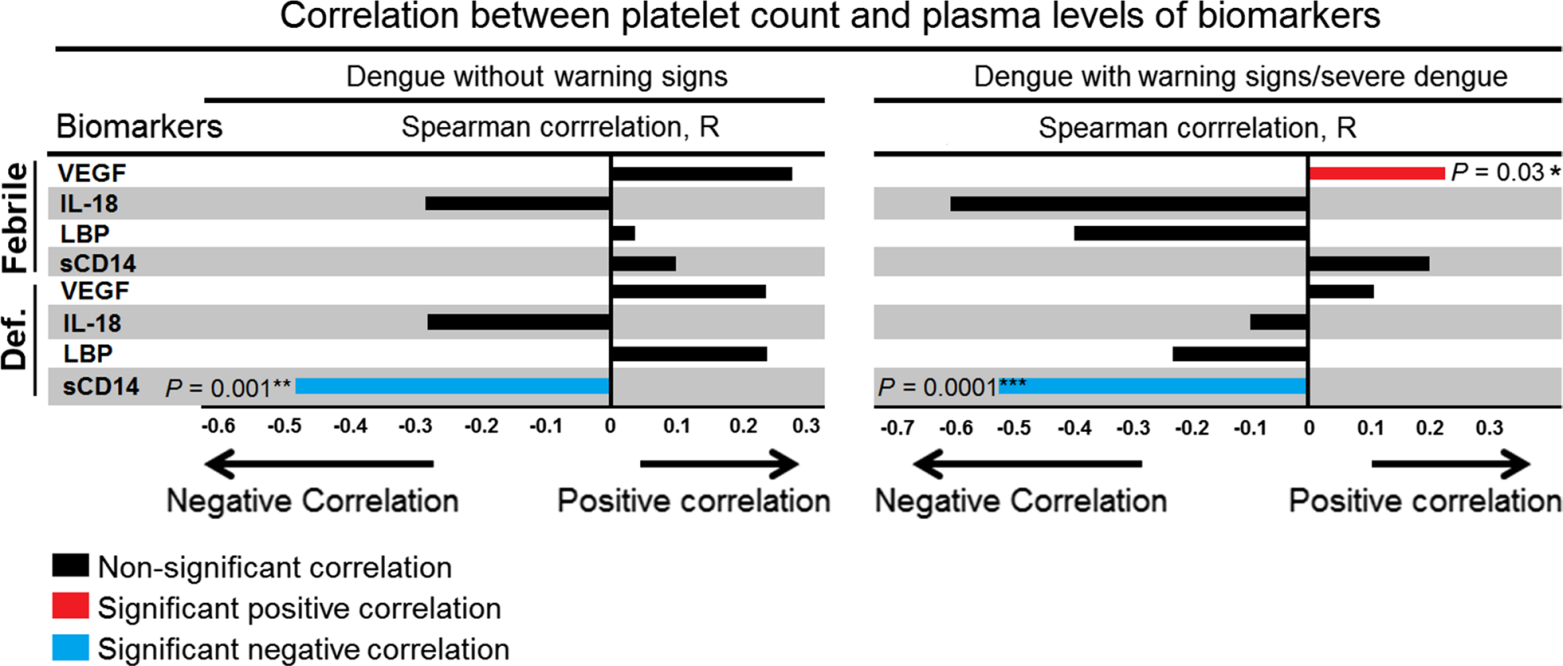
Spearman correlations between platelet count and plasma levels of biomarkers in *left panel* dengue patient without warning signs, and *right panel* dengue patient with warning signs and severe dengue. The *bar* represents the strength of association (r values); *bars* point to the *right* for a positive association and to the *left* for a negative association. The *red bar* represents significant positive correlation, *blue bar* represents significant negative correlation and *black bar* represent non-significant correlation. *P* value less than 0.05 (significant association), where ****<0.0001, ***<0.001, **<0.01 and <0.05

Since thrombocytopenia is observed for dengue haemorrhagic fever, these cytokines were investigated for any association with platelet counts. At febrile phase, Spearman correlation analysis showed that VEGF positively correlated with platelet counts in patient with SD and DWS+ (r = 0.228, *P* = 0.03); whilst sCD14 showed significant inverse correlation with platelet counts at defervescence phase in both DWS− (r = −0.483, *P* = 0.023) and DWS+ (r = −0.473, *P* = 0.023) groups (Fig. 5). Linear regression model showed that with every increase of 100 pg/ml of sCD14, an association with a decrease in platelet count by 1.6, 95% CI (−2.4, −0.94), *P* < 0.0001 was noted. This suggests that sCD14 may have a mediatory role on blood vassel permeability/thrombocytopenia.

## Discussion

Dengue has become a public health concern. With the new dengue classification by WHO in 2009, studies in the past that predict DHF and DSS according to the WHO 1997 classification may not be applicable to this new classification. Early prediction of DWS+ and SD is of pivotal importance towards identifying dengue patients who will need the best supportive care, thereby helping to optimize the management of severe cases [12]. An ideal biomarker needs to identify individuals who are at risk of developing DWS+ or SD when they first present at the hospital, typically during the febrile stage. The present study is the first to investigate the association of biomarkers using the new WHO classification. We investigated 20 plasma biomarkers and identified that IL-18 at both febrile and defervescence phases as well as LBP and sCD14 at febrile phase are of the best predictive value. In addition, decreased levels of IP-10 and IL-10 as well as increased levels of CXCL6 and VEGF were associated with development of SD. We also show that the platelet counts were correlated with VEGF and inversely correlated with sCD14.

Most hypotheses explaining dengue immunopathogenesis suggest that the overproduction and/or a skewed cytokine response during the critical phase of disease causes plasma leakage and hence, a more severe manifestation of dengue [8]. Here we observed that the severity of dengue disease was associated with aberrant activation of monocyte or macrophages as evidenced by elevation of plasma IL-18, sCD14 and LBP particularly at the febrile phase. Furthermore, the free circulating IL-18 remained significantly elevated among patients with the more severe form of dengue disease.

IL-18 is a potent proinflammatory cytokine predominantly produced by activated monocytes/macrophages via the NLRP3 inflammasome pathway [32, 33]. Interleukin-18 is present in monocytes or macrophages as a biologically inactive precursor form, proIL-18 [32, 33]. Upon NLRP3 activation and assembly, the proIL-18 will be further processed by intracellular cysteine protease caspase-1 and released as a fully functional matured IL-18. The elevation of IL-18 among DWS+ and SD patients in this cohort suggest that aberrant activation of the inflammasome may play a role in dengue immunopathogenesis, consistent with previous reports [7, 17, 34, 35]. IL-18 secreted by monocytes/macrophages will then induce activation and expression of both CC and CXC chemokines from a wide range of cells [36] enhancing natural killer (NK) cell cytotoxicity, neutrophil activity, and IFN-γ production by T cells and NK cells [36]. This is in line with our observation that CXCL6, a neutrophilic chemokine was elevated among DWS+ and SD patients at the defervescence phase.

However, the biological activity of IL-18 is regulated by a naturally occurring IL-18 binding protein (IL-18BP). To gain better insight into the regulation of biological activity of IL-18 during dengue disease, we measured the plasma levels of IL-18BPa and free circulating IL-18 levels were estimated. Our data demonstrated that free circulating IL-18 was associated with severity of the dengue disease. Furthermore, the correlation between IL-18 and IL-18BP was stronger in DWS− patients as compared to DWS+/SD patients. In contrast, Michels et al. [37] showed no significant elevation of free circulating IL-18 among patients with severe dengue as compared to non-severe dengue. The exact reason behind this discrepancy is not known and may reflect the use of different assay methods or differences in patients’ characteristics and therefore require further investigation. Despite this, other studies have also found that dengue virus is capable to induce inflammasome activation via CLEC5A [38]. Furthermore, NLRP-3 inflammasome in platelet was shown to be activated after expose to dengue virus in vitro and the level of inflammasome activation correlates with vascular permeability [39]. These findings suggest that inflammasome activation may play a significant role in the immunopathogenesis of dengue disease.

Microbial translocation (MT) is a phenomenon characterized by translocation of microbial products e.g. LPS from the gut into blood stream under inflammatory conditions. Although MT has been studied extensively in HIV disease [40], it’s also shown to play an important role in other diseases such as graft versus host disease after hematopoietic stem cell transplantation [41], inflammatory bowel syndrome [42], chronic liver disease [43] and even end stage of kidney disease [44]. Recently, MT has been observed among dengue patients [25] and levels of LPS was associated with severity of dengue disease [26]. Extending from these study, we sought to determine the values of MT markers i.e. LBP and sCD14 in predicting severity of dengue disease. The LBP is a soluble acute-phase protein that binds to bacterial LPS and triggers immune responses by transferring the LPS to sCD14 and TLR4 expressed on cell surface of monocyte/macrophages [45], while sCD14 is secreted by monocyte and macrophages upon LPS stimulation [46]. While our data showed that both LBP and sCD14 were predicted with DWS+ and SD, consistent with previous reports [25, 47], by using a multivariate regression model, we showed IL-18, LBP and sCD14 were independently associated with increased risk of developing DWS+ and SD. Furthermore, we also showed sCD14 was inversely correlated with platelet counts. This data suggests that apart from inflammasome activation, MT also plays an important role in driving the exacerbated inflammation in dengue disease. Since LPS is also known for causing systemic immune activation and noncanonical inflammasome activation, thus elevation of plasma LPS among DWS+ and SD patients is thereby fuelling inflammatory responses.

Although DWS+ is associated with some forms of plasma leakage and spontaneous bleeding, this does not invariably lead to SD which is characterised by severe plasma leakage leading to the reduced pulse pressure, disseminated intravascular coagulation and multiple organ failure. We therefore sought to identify possible cytokines and immune factors that may contribute to the development of severe dengue. Our data showed that SD was associated with decreased levels of IL-10 and IP-10 and increased levels of CXCL6 at the defervescence phase. One study found that patients who developed severe dengue had significantly lower T cell counts when compared to non-severe dengue, whilst their serum IL-10 and IP-10 levels positively correlated with T cell apoptosis [48]. Therefore, it is plausible that the decreased of IL-10 and IP-10 among patients with SD in our cohort was due to excessive loss of T cells. CXCL6 is a chemokine that is known to exert potent neutrophil activation and chemotaxis [49], hence elevation of CXCL6 and VEGF among SD patients may suggest a role for neutrophils in the pathogenesis of SD.

To date, the causes of increased vascular permeability among dengue patients is not well understood. Several lines of evidence suggest that endothelial dysfunction rather than necrosis of the endothelium is likely to be the cause of vascular leak [50, 51]. VEGF is a potent growth factor that has a role in promoting endothelial permeability and proliferation and it may contribute to inflammation and coagulation [52]. Consistent with previous reports [8, 53, 54], we found that the VEGF was consistently elevated at both febrile and defervescence phases. Since VEGF is a platelet derived growth factor [55], hence elevation of VEGF among patients with severe dengue may also suggest aberrant platelet activation.

One limitation in our study was the sample size number especially in the severe dengue group as there were only six of them in the febrile phase and 4 in the defervescence phase, hence losing out some important details as we had to combine the SD patients with DWS+ patients in some of the analysis. Future study with bigger numbers of SD patients is warranted. Notwithstanding this limitation, our study has identified some plasma markers to allow for early and accurate prediction of DWS+ and SD, thereby serving as possible biomarkers especially during outbreaks.

## Conclusions

The present study identifies IL-18, LBP and sCD14 as robust early predictors for identifying patients who may be at risk of developing DWS+ and SD. Given that the elevation of IL-18, sCD14 and LBP were elevated among patients with severe dengue disease and the levels of sCD14 inversely correlated with platelet counts, our findings suggest a pivotal role of microbial translocation and an aberrant activation of the inflammasome in the development of severe form of dengue disease.

## Additional files

**Additional file 1: Table S1.** The criteria for dengue with or without warning signs and severe dengue. **Table S2.** Lowest detection limit and dilution factor for each analyte.

**Additional file 2: Figure S1.** **(A)** Plasma levels IL-18BPa and free circulating IL-18 in dengue patients at febrile and defeverscence phase. **(B)** Spearman correlation between IL-18 and IL-18BP among DWS and DWS+/SD. Levels of biomarkers were compared across the three patient groups and post hoc Mann–Whitney U tests were then performed for those biomarkers with a Kruskal–Wallis test *P* value of <0.05. A Spearman rank test was used to compare the correlation between two continuous variables. ****P < 0.0001, ***P < 0.001, **P < 0.01, and *P < 0.05.

## Abbreviations

DENV: dengue virus
DHF: dengue haemorrhagic fever
DSS: dengue shock syndrome
DWS−: dengue without warning signs
DWS+: dengue with warning signs
SD: severe dengue
LPS: lipopolysaccharide
LBP: LPS binding protein
DAMPs: danger-associated molecule patterns
sCD14: soluble CD14
sCD163: soluble CD163
sCD25: soluble CD25
IL-10: interleukine-10
IL-18: interleukine-18
IFN-γ: interferon-gamma
HGF: hepatocyte growth factor
MFI: macrophage migration inhibitory factor
MCP-1: monocyte chemoattractant protein-1
MIP-1b: macrophage inflammatory proteins-1b
VEGF: vascular endothelial growth factor
ICAM-1: intercellular adhesion molecule 1
VCAM-1: vascular cell adhesion molecule 1
ROC: receiver operating characteristic
CI: confidence interval
IQR: interqualtile range
