# Supplementary material for: Aberrant monocyte responses predict and characterize dengue virus infection in individuals with severe disease
Source: J Transl Med. 2017 May 31;15:121. doi: 10.1186/s12967-017-1226-4 (PMC5452397; doi:10.1186/s12967-017-1226-4)
Supplement: Supplementary file 1 — Additional file 1: Table S1. The criteria for dengue with or without warning signs and severe dengue. Table S2. Lowest detection limit and dilution factor for each analyte. [file 12967_2017_1226_MOESM1_ESM.docx]

**Supplementary Table S1: The criteria for dengue with or without warning signs and severe dengue.**

| Categories | Dengue without warning signs | Warning signs | Severe dengue |
| --- | --- | --- | --- |
| **Characteristics** | - Nausea, vomiting - Rash - Aches and pains - Leukopenia - Tourniquet test positive | - Abdominal pain - Persistent vomiting - Clinical fluid accumulation - Mucosal bleeding - Lethargy, restlessness - Liver enlargement >2cm - Increase in HCT concurrent with rapid decrease in platelet count | Severe plasma leakage:   - Shock (DSS) - Fluid accumulation with respiratory distress   Severe organ involvement:   - Liver: AST or ALT ≥ 1000 - CNC: Impaired consciousness - Heart and other organs |

**Supplementary Table S2: Lowest detection limit and dilution factor for each analyte**

|  | **Biomarker** | **Lowest detection limit (pg/ml)** | **Dilution factor** | **Assay type** |
| --- | --- | --- | --- | --- |
|  | **Inflammation pathway** | | |  |
| 1 | sCD25 | 20.5 | 1:4 | Luminex screening assay |
| 2 | IFN-γ | 102 | 1:4 | Luminex screening assay |
| 3 | Granzyme B | 22.3 | 1:4 | Luminex screening assay |
| 4 | IL-10 | 13.5 | 1:4 | Luminex screening assay |
| 5 | HGF | 40.7 | 1:4 | Luminex screening assay |
| 6 | IL-18 | 10.24 | 1:5 | Quantikine ELISA kit |
|  | IL-18BPa | 26.6 | 1:10 | Quantikine ELISA kit |
| 7 | sCD163 | 4200 | 1:4 | Luminex screening assay |
| 8 | CXCL6 | 28 | 1:4 | Luminex screening assay |
| 9 | CXCL8 | 8 | 1:4 | Luminex screening assay |
| 10 | CX3CL1 | 1438 | 1:4 | Luminex screening assay |
| 11 | IP-10 | 2.5 | 1:4 | Luminex screening assay |
| 12 | MCP-1 | 32 | 1:4 | Luminex screening assay |
| 13 | MIP-1b | 86.8 | 1:4 | Luminex screening assay |
| 14 | RNTES | 15.6 | 1:5 | Quantikine ELISA kit |
| 15 | MIF | 15.6 | 1:5 | Quantikine ELISA kit |
|  | **Endothelial activation pathway** | | |  |
| 16 | ICAM-1 | 7400 | 1:4 | Luminex screening assay |
| 17 | VCAM-1 | 7725 | 1:4 | Luminex screening assay |
| 18 | VEGF | 13 | 1:4 | Luminex screening assay |
|  | **Microbial translocation pathway** | | |  |
| 19 | LBP | 3130 | 1:800 | Quantikine ELISA kit |
| 20 | sCD14 | 250 | 1:200 | Quantikine ELISA kit |
