# Supplementary material for: Aberrant monocyte responses predict and characterize dengue virus infection in individuals with severe disease
Source: J Transl Med. 2017 May 31;15:121. doi: 10.1186/s12967-017-1226-4 (PMC5452397; doi:10.1186/s12967-017-1226-4)
Supplement: Supplementary file 2 — Additional file 2: Figure S1. (A) Plasma levels IL-18BPa and free circulating IL-18 in dengue patients at febrile and defeverscence phase. (B) Spearman correlation between IL-18 and IL-18BP among DWS and DWS+/SD. Levels of biomarkers were compared across the three patient groups and post hoc Mann–Whitney U tests were then performed for those biomarkers with a Kruskal–Wallis test P value of <0.05. A Spearman rank test was used to compare the correlation between two continuous variables. ****P < 0.0001, ***P < 0.001, **P < 0.01, and *P < 0.05. [file 12967_2017_1226_MOESM2_ESM.docx]

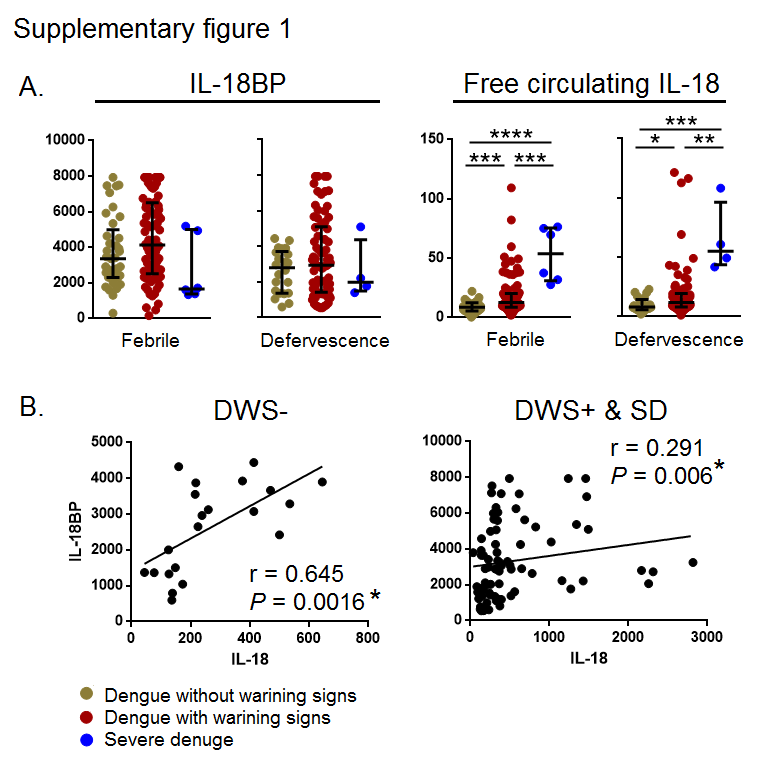


**Supplementary Figure S1. (A)** Plasma levels IL-18BPa and free circulating IL-18 in dengue patients at febrile and defeverscence phase. **(B)** Spearman correlation between IL-18 and IL-18BP among DWS and DWS+/SD. Levels of biomarkers were compared across the three patient groups and post hoc Mann–Whitney U tests were then performed for those biomarkers with a Kruskal–Wallis test *P* value of < 0.05. A Spearman rank test was used to compare the correlation between two continuous variables. *****P* < 0.0001, ****P* < 0.001, ***P* < 0.01, and **P* < 0.05.
